# Comparative effects of left bundle branch area pacing, His bundle pacing, biventricular pacing in patients requiring cardiac resynchronization therapy: A network meta‐analysis

**DOI:** 10.1002/clc.23784

**Published:** 2022-02-07

**Authors:** Juan Hua, Chenxi Wang, Qiling Kong, Yichu Zhang, Qijun Wang, Ziyi Xiong, Jinzhu Hu, Juxiang Li, Qi Chen, Kui Hong

**Affiliations:** ^1^ Department of Cardiology The Second Affiliated Hospital of Nanchang University Nanchang China

**Keywords:** biventricular pacing, cardiac resynchronization therapy, His bundle pacing, left bundle branch area pacing, network meta‐analysis

## Abstract

**Background:**

The comparative effects of different types of cardiac resynchronization therapy (CRT) delivered by biventricular pacing (BVP), His bundle pacing (HBP), and left bundle branch area pacing (LBBAP) remain inconclusive.

**Hypothesis:**

HBP and LBBAP may be advantageous over BVP for CRT.

**Methods:**

PubMed, Embase, Web of Science, and the Cochrane Library were systematically searched for studies that reported the effects after BVP, HBP, and LBBAP for CRT. The effects between groups were compared by a frequentist random‐effects network meta‐analysis (NMA), by which the mean differences (MDs) and 95% confidence intervals (CIs) were calculated.

**Results:**

Six articles involving 389 patients remained for the final meta‐analysis. The mean follow‐up of these studies was 8.03 ± 3.15 months. LBBAP resulted in a greater improvement in LVEF% (MD = 7.17, 95% CI = 4.31 to 10.04), followed by HBP (MD = 4.06, 95% CI = 1.09 to 7.03) compared with BVP. HBP resulted in a narrower QRS duration (MD = 31.58 ms, 95% CI = 12.75 to 50.40), followed by LBBAP (MD = 27.40 ms, 95% CI = 10.81 to 43.99) compared with BVP. No significant differences of changes in LVEF improvement and QRS narrowing were observed between LBBAP and HBP. The pacing threshold of LBBAP was significantly lower than those of BVP and HBP.

**Conclusion:**

The NMA first found that LBBAP and HBP resulted in a greater LVEF improvement and a narrower QRS duration compared with BVP. Additionally, LBBAP resulted in similar clinical outcomes but with lower pacing thresholds, and may therefore offer advantages than does HBP for CRT.

## 1 INTRODUCTION

Heart failure (HF) remains to be a serious public health concern, with high mortality, morbidity, and poor quality of life.^1^ Cardiac resynchronization therapy (CRT) is effective for HF, particularly in those with reduced systolic heart failure and either bundle branch block (BBB) or need for frequent ventricular pacing.^2^, ^3^ The clinical benefits of CRT delivered by biventricular pacing (BVP) are remarkable.^4^, ^5^ However, approximately 30% of patients do not respond to CRT with BVP. In fact, BVP results in the fusion of two nonphysiological wave fronts and leaves a substantial degree of residual dyssynchrony.^6^, ^7^ Moreover, the success rate of BVP for CRT was about 95.9%, and the complication incidence was 7%–10%.^8^, ^9^ Computer modeling indicates that there would be more possibilities to improve cardiac functionality when greater ventricular resynchronization could be achieved.^10^

His bundle pacing (HBP) has become a possible alternative for CRT with physiological restoration of normal physiologic His‐Purkinje conduction and promoted superior electrical ventricular resynchronization than BVP.^6^, ^11^, ^12^ Moreover, several other studies confirmed that HBP corrected the left bundle branch block (LBBB) by pacing the distal portions of the His bundle (His‐CRT).^13^, ^14^ When successful, this contributes to a normality of LV electrical activation and thereby a more “physiological” correction of dyssynchrony. Nevertheless, HBP has some shortcomings and limits its application, such as low R‐wave amplitude, high pacing thresholds, and technical difficulties.^15^ The average implant success rate of HBP was 84.8%, and the complication incidence was about 4.7%.^16^

In 2017, Huang et al.^17^ first conceived the left bundle branch pacing (LBBP) and demonstrated that it delivered clinical benefits in a patient with HF and LBBB, which targets pacing the proximal left bundle branch and its branches along with capture of LV septal myocardium. Selective LBBP (S‐LBBP) only captures the LBB without myocardial capture, while nonselective LBBP (NS‐LBBP) captures both the LBB and the local myocardium.^18^ It is defined as left ventricular septal pacing (LVSP) if only LV septal myocardium is captured.^18^ Left bundle branch area pacing (LBBAP), with the lead implanted slightly distal to the His bundle and screwed deep in the LV septum ideally to capture LBB, which means LBBP or LVSP, without clear evidence for LBB capture.^19^ Subsequently, several case reports and observational studies demonstrated the effectiveness and safety of LBBAP in patients requiring CRT during short‐ and mid‐term follow‐up.^20^, ^21^, ^22^, ^23^ Furthermore, the success rate of LBBAP was reported varied from 90.9% to 97.8%,^24^, ^25^, ^26^ and the overall complication incidence of procedure‐related and long‐term follow‐up was about 1.6%–2.8%.^26^, ^27^ However, only a few studies compared the feasibility and effects of these different types of CRT delivered by BVP, HBP, and LBBAP, especially direct comparison between HBP and LBBAP. Thus, we aimed to systematically review the studies of BVP, HBP, and LBBAP for CRT to perform a network meta‐analysis of existing data.

## 2 METHODS

### 2.1 Literature review and search strategy

All search results were assessed in accordance with the PRISMA guidelines.^28^ A systematic literature search of PubMed, Embase, Web of Science, and the Cochrane Library was conducted to compare the following outcomes: changes in left ventricular ejection fraction (LVEF) between BVP, HBP, and LBBAP for CRT. Two investigators (Juan Hua and Chenxi Wang) conducted a systematic literature review independently. The search was performed with keywords as follows: “Cardiac resynchronization therapy,” “Biventricular pacing,” “His bundle pacing,” and “Left bundle branch pacing” or “Left ventricular septal pacing” or “Left bundle branch area pacing,” alone and in combination. The search strategies were shown in Table S1.

### 2.2 Selection criteria

Articles reporting HBP or LBBAP in patients undergoing CRT were included in the English language. The *PICOS* (Populations, Interventions, Comparisons, Outcomes, and Study design) criteria of our study were as follows: *Populations*: Advanced HF requiring CRT; *Interventions*: CRT was delivered by BVP, HBP, or LBBAP; *Comparisons*: BVP versus HBP, BVP versus LBBAP, or BVP versus HBP versus LBBAP; *Outcomes*: Changes in LVEF improvement, changes in QRS duration (QRSd) narrowing, pacing threshold of His lead or LV lead; and *Study design*: randomized controlled trials (RCTs), or observational studies. Review articles, case reports, editorials/letters, abstracts, and studies with patients <10 were excluded. The full texts of all potentially relevant articles were assessed for compliance with the inclusion and exclusion criteria. Two reviewers (Qiling Kong and Yichu Zhang) screened the selected articles independently based on the title and abstract. Any discordance was settled through discussion between the reviewers. The pacing threshold was the His lead for HBP and LBBAP and LV lead for BVP at implantation or a week after implantation at 0.4, 0.5, or 1.0 ms.

### 2.3 Data abstraction and quality assessment

Data from each enrolled article were independently extracted by two reviewers (Qijun Wang and Ziyi Xiong). Background information such as authors, years, the region of trial, indication, intervention, duration of follow‐up, and outcomes were extracted from each article. All conflicts were resolved through discussion between the reviewers. The Cochrane Handbook for Systematic Reviews of Interventions (version 5.4.0) was used to evaluate the quality of the selected RCTs.^29^ Observational studies were evaluated using the Newcastle‐Ottawa Scale.^30^ Studies with six or more points were regarded as having a high quality.

### 2.4 Statistical analyses

Network meta‐analysis using a network analysis tool that combined direct and indirect evidence in a mixed‐intervention model was performed.^31^ For each interest outcome, the effect measurement estimated chosen for the continuous variables were the mean differences (MDs) and their corresponding 95% confidence intervals (CIs). The surface under the cumulative ranking area (SUCRA) probabilities were selected to calculate the ranking and hierarchy of the different treatments.^31^ The larger SUCRA indicates the greater probability of becoming the best intervention. The network meta‐analysis was conducted using the frequentist methods with restricted maximum likelihood estimation to quantify network heterogeneity and to assume a common heterogeneity estimate within a network. In addition, the local inconsistencies across studies in each closed loop were evaluated using the node splitting approaches.^32^ Publication bias was evaluated using the funnel plots. These analyses were conducted using the Stata version 15.0 (Stata Corp).

## 3 RESULTS

### 3.1 Study selection and quality assessment

**Figure 1:**
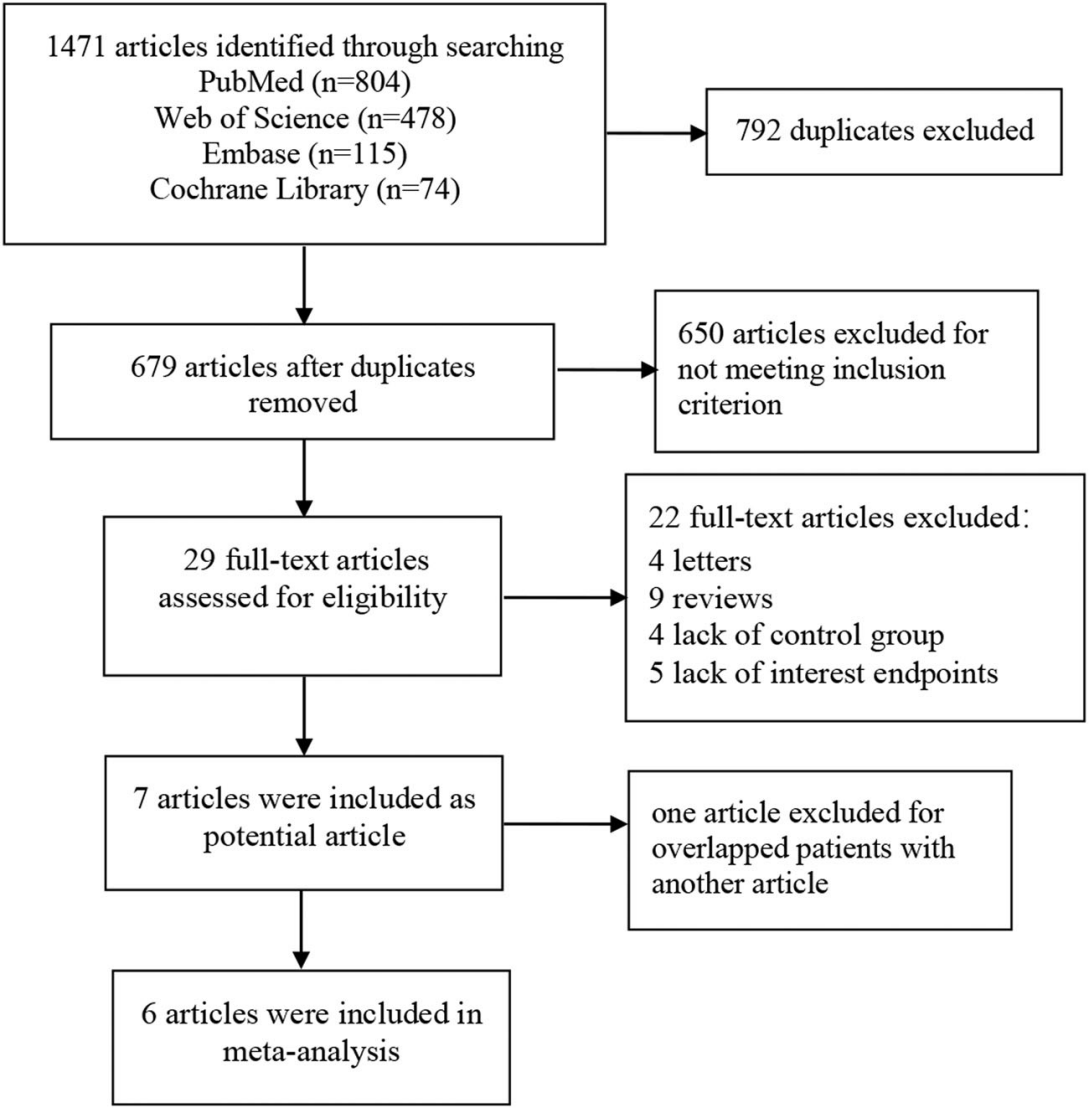
A flow diagram of the included studies. The literature search yielded 1471 articles through searches of PubMed, Embase, Web of Science, and the Cochrane Library databases. After searching for compliance with the inclusion and exclusion criteria, six articles were selected for this final meta‐analysis

The literature search yielded 1471 articles (804 from PubMed, 478 from Web of Science, 115 from Embase, and 74 from the Cochrane Library), which were considered as potential studies. After the removal of duplicates, 679 publications remained. Afterward, 650 articles were excluded by preliminary screening of titles and abstracts, and a total of 29 were further evaluated comprehensively. Then, 22 records were excluded because of various reasons. Seven articles were found to be eligible for the present meta‐analysis after a full‐text review.^11^, ^21^, ^23^, ^33^, ^34^, ^35^, ^36^ However, two included studies were from the same center, the patients were included from December 2012 to December 2018,^35^ and January 2012 to June 2017,^11^ respectively. It seems that some patients included were overlapped, so we determined to delete the study of Huang et al, which included relatively few patients.^11^ Finally, six studies were selected for the present analysis. The selection process for the literature included in the analysis was shown in Figure 1. The basic characteristics, demographics of the study participants, and the quality evaluation of observational studies were shown in Table 1. The mean follow‐up of these studies was 8.03 ± 3.15 months. RCTs were evaluated by Cochrane Handbook for Systematic Reviews of Interventions and shown in Figure S1. The network plots for comparisons of outcomes were shown in Figure S2.

**Table 1:** Characteristics of included studies

Authors	Year	Regions	Study design	Total patients	Study patients	Interventions	Follow‐up (months)	Outcomes	NOS
Guo et al.^21^	2020	China	Non‐RCT	42	LBBB morphology (Strauss's criteria), with LVEF ≤ 35%, NYHA Classes II–IV	BVP versus LBBAP	6	LVEF, LVEDD, LVESD, NYHA class, QRSd, pacing threshold	7
Li et al.^23^	2020	China	Non‐RCT	81	LBBB and LVEF ≤ 35%	BVP versus LBBAP	6	LVEF, LVEDD, QRSd, HYHA class, NT‐pro BNP, pacing threshold	7
Upadhyay et al.^33^	2019	Chicago	RCT	41	NYHA II–IV patients with QRS ＞ 120 ms	BVP versus HBP	12.2	QRSd, LVEF, freedom from CV hospitalization and mortality	a
Wang et al.^34^	2020	China	Non‐RCT	40	SR, CLBBB with QRSd > 140 ms (M) and >130 ms (F), LVEF ≤ 35%, and NYHA classes II–IV	BVP versus LBBAP	6	QRSd, LVEF, LVEDD, LVESD, LVEDV, LVESV, NYHA class and BNP, pacing threshold	7
Wu et al.^35^	2020	China	Non‐RCT	135	LVEF ≤ 40% and typical LBBB	BVP versus HBP versus LBBAP	12	LVEF, QRSd, pacing threshold, NYHA class	8
Vinther et al.^36^	2021	Denmark	RCT	50	Symptomatic HF, LVEF ≤ 35% and LBBB	BVP versus HBP	6	QRSd, LVEF, LVESV, NYHA class, NT‐pro BNP, pacing threshold	a

Abbreviations: BNP, B‐type natriuretic peptide; BVP, biventricular pacing; CLBBB, complete left bundle branch block; CRT, cardiac resynchronization therapy; CV, cardiovascular; HBP, His bundle pacing; HF, heart failure; LBBAP, left bundle branch area pacing; LBBB, left bundle branch block; LVEDD, left ventricular end‐diastolic diameter; LVEDV, left ventricular end‐diastolic volume; LVEF, left ventricular ejection fraction; LVESD, left ventricular end‐systolic dimension; LVESV, left ventricular end‐systolic volume; M, man; F, female; Non‐RCT, nonrandomized controlled trial; NOS, Newcastle‐Ottawa Scale; NYHA, New York Heart Association; QRSd, QRS duration; RCT, randomized controlled trial; SR, sinus rhythm.

^a^ RCTs were evaluated by Cochrane handbook for systematic reviews of interventions (shown in Figure S1).

### 3.2 Changes in LVEF improvement

**Figure 2:**
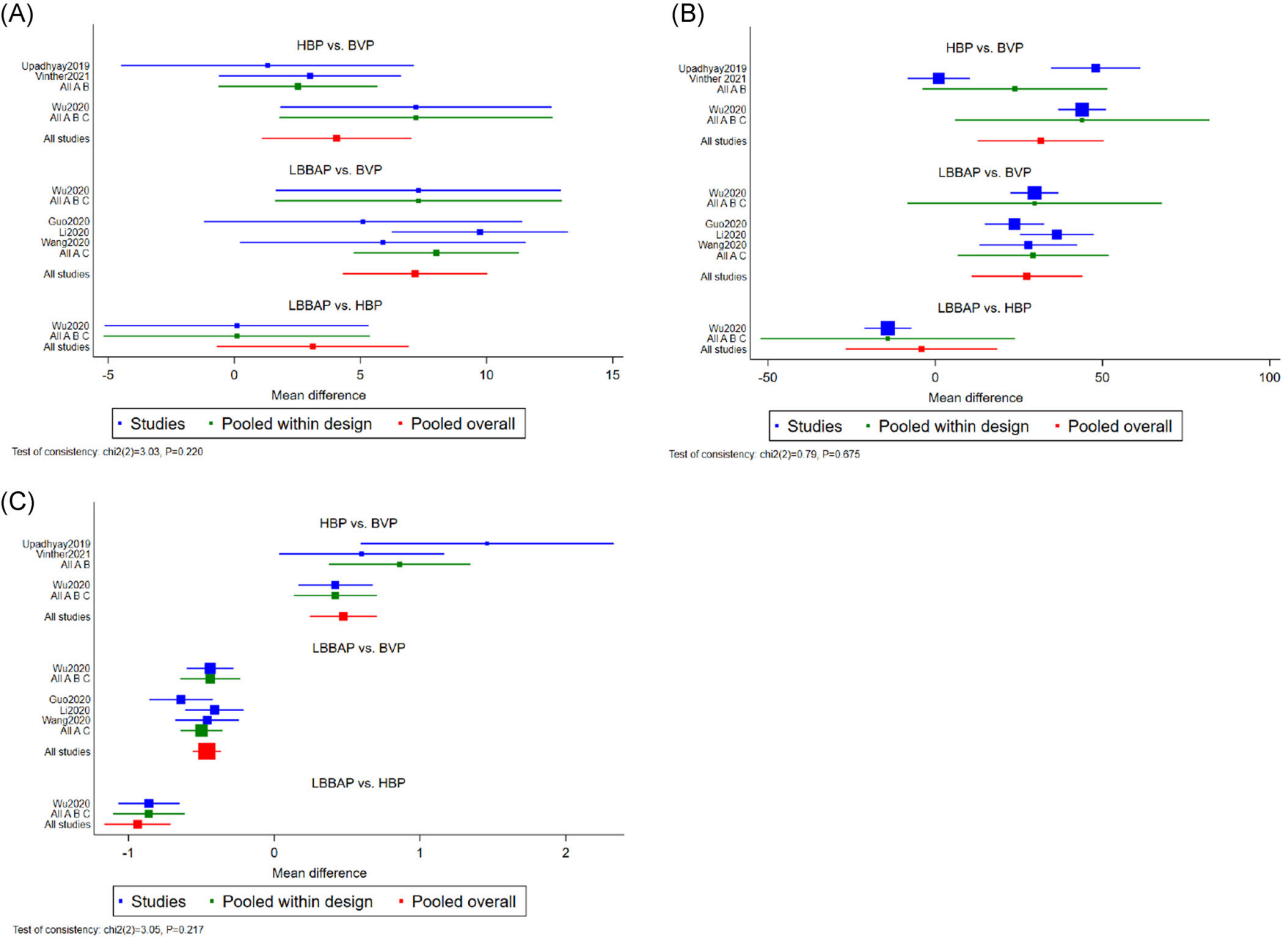
Forrest plot demonstrating changes in LVEF improvement (A), changes in QRSd narrowing (B), and pacing threshold (C) between BVP, HBP, and LBBAP. Square data markers represented the MDs of the different outcomes. The horizontal lines represented the 95% CIs. BVP, biventricular pacing; CIs, confidence intervals; HBP, His bundle pacing; LBBAP, left bundle branch area pacing; LVEF, left ventricular ejection fraction; MDs, mean differences; QRSd, QRS duration

**Figure 3:**
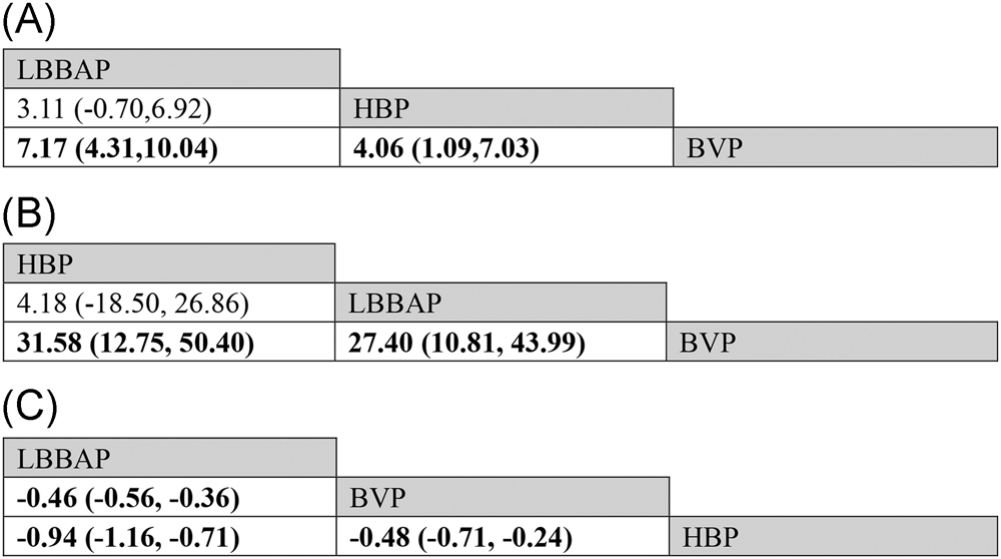
League table (MD [95% CI]) of changes in LVEF improvement (A), changes in QRSd narrowing (B), and pacing threshold (C) between BVP, HBP, and LBBAP. Significant differences were highlighted by bold type. Abbreviations were as in Figure 2

**Figure 4:**
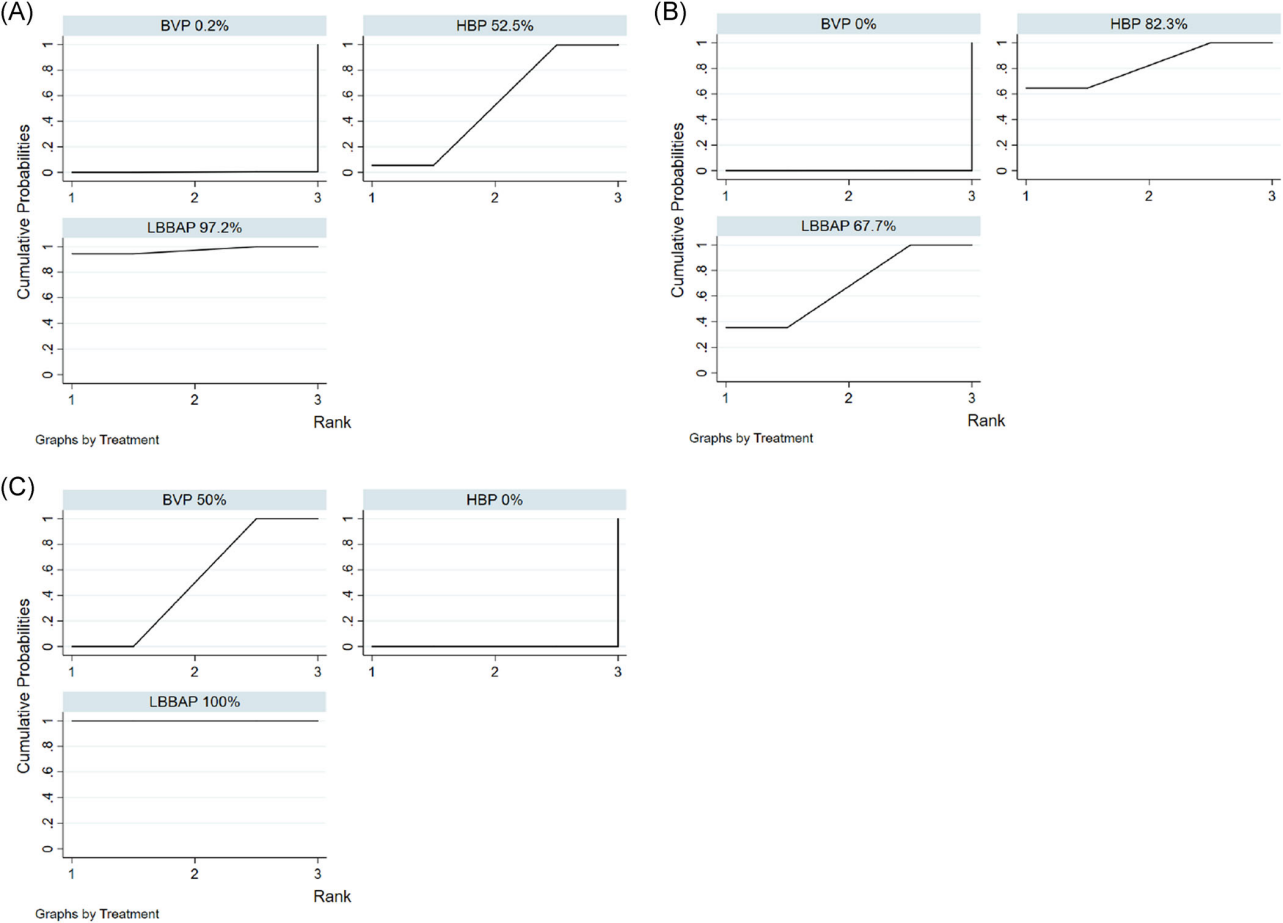
The SUCRA represented the overall ranking effect of changes in LVEF improvement (A), changes in QRSd narrowing (B), and pacing thresholds (C) between BVP, HBP, and LBBAP. A large SUCRA value corresponds to a high probability of the interest endpoint event. SUCRA, surface under the cumulative ranking area. Other abbreviations were as in Figure 2

All articles selected involving a total of 375 subjects who reported changes in LVEF, including 203 patients for BVP, 84 for HBP, and 88 for LBBAP. When compared to BVP, LBBAP resulted in the greatest LVEF% improvement with a MD of 7.17 (95% CI = 4.31 to 10.04), followed by HBP with a MD of 4.06 (95% CI = 1.09 to 7.03) (Figure 2A). In addition, no statistical difference was observed in improvement of LVEF between LBBAP and HBP (MD = 3.11, 95% CI = −0.70 to 6.92). League table for changes in LVEF was shown in Figure 3A. Regarding changes in LVEF improvement, LBBAP (SUCRA 97.2%) was the best treatment, followed by HBP (SUCRA 52.5%) and BVP (SUCRA 0.2%) (Figure 4A).

### 3.3 Changes in QRS duration narrowing

All articles selected involving a total of 388 subjects who reported changes in QRSd, including 208 patients for BVP, 90 for HBP, and 90 for LBBAP. When compared to BVP, HBP resulted in narrower QRS duration with a MD of 31.58 ms (95% CI = 12.75 to 50.40), followed by LBBAP with a MD of 27.40 ms (95% CI = 10.81 to 43.99) (Figure 2B). In addition, no statistical difference was observed in the QRSd narrowing between LBBAP and HBP (MD = −4.18 ms, 95% CI = −26.86 to 18.50). League table for changes in QRSd was shown in Figure 3B. Regarding changes in QRS narrowing, HBP (SUCRA 82.3%) was the best treatment, followed by LBBAP (SUCRA 67.7%) and BVP (SUCRA 0%) (Figure 4B).

### 3.4 Pacing threshold

All articles selected involving 388 subjects and reported pacing thresholds, including 208 patients for BVP, 90 for HBP, and 90 for LBBAP. When compared to BVP, LBBAP patients had a lower pacing threshold with a MD of −0.46 V (95% CI = −0.56 to −0.36). Conversely, HBP patients had a higher pacing threshold with a MD of 0.48 V (95% CI = 0.24 to 0.71) compared with BVP (Figure 2C). In addition, the pacing threshold in LBBAP was significantly lower than HBP with a MD of −0.94 V (95% CI = −1.16 to −0.71). League table for pacing threshold was shown in Figure 3C. Regarding the pacing threshold, LBBAP (SUCRA 100%) was the best treatment, followed by BVP (SUCRA 50%) and HBP (SUCRA 0%) (Figure 4C).

### 3.5 Exploration of inconsistency and publication bias

No inconsistency was found to be in both global (Figure S3) and local tests (Tables S2–S4) in this NMA. No significant publication bias but changes in QRS duration was found in the funnel plot (Figure S4).

## 4 DISCUSSION

Several case reports and single‐center studies that explored the use of the HBP and LBBAP for CRT have been published. The sample sizes in the published studies were relatively small, and the comparisons across them were limited, especially between HBP and LBBAP. Therefore, we aimed to systematically review the literature on HBP or LBBAP for CRT and conduct a network meta‐analysis (NMA) of the available data. Notably, this is the first NMA on the topic of CRT delivered by BVP, HBP, and LBBAP. One advantage of this NMA was to indirectly compare the head‐to‐head of the three types of pacing interventions simultaneously. Another advantage of this NMA was the calculation of the ranking and hierarchy of these treatments for CRT. In this study, we found that HBP and LBBAP delivered greater LVEF improvement and narrower QRS duration than BVP. Additionally, the pacing threshold of LBBAP was significantly lower than those of BVP and HBP.

CRT is recommended for symptomatic patients with HF in sinus rhythm with a QRS duration ≥150 ms and in those with LBBB QRS morphology with an LVEF ≤ 35% despite optimized medical treatment to improve symptoms and reduce morbidity and mortality, with Class I recommendation of the 2019 American College of Cardiology/American Heart Association (ACC/AHA) and European Society of Cardiology (ESC) heart failure guidelines.^37^ All patients of the included studies in our meta‐analysis were almost in accordance with the guidelines for CRT. There have been several alternative types of CRT delivery, such as LV endocardial pacing, HBP, and LBBAP. Several studies have demonstrated that CRT by BVP improved cardiac functionality and long‐term survival by reducing cardiac workload and HF hospitalizations.^38^, ^39^, ^40^, ^41^ However, the response to BVP differs significantly and ranges from complete normalization of cardiac function to no response.

The His‐Purkinje system pacing is currently considered to be the optimal physiologic pacing technique, with the pacing lead directly implanted in the conduction system to narrow the QRS complex and improve cardiac function by selective or nonselective HBP.^11^, ^42^ Nevertheless, there are several limitations with HBP, which may restrict its wide clinical application, such as high corrective thresholds and late threshold increases. LBBAP, as a novel pacing technique, aims to correct the LBB conduction system desynchrony therefore deliver satisfactory LV synchrony and immediate hemodynamic benefits.^43^ Meanwhile, it has a lower and stable pacing threshold and a physiological pacing site to prevent the occurrence of conduction disorders.^26^ Furthermore, LBBAP is associated with high success rate and low complication incidence.^44^

The QRS duration has been identified as a powerful prognostic marker, and its significance is well known in patients with heart failure.^45^ A QRS complex ≥120 ms results in a more advanced myocardial disease, worse prognosis, and higher all‐cause mortality.^46^ The QRS duration is an established predictor of response to CRT,^47^ and its changes from before to after pacing are also considered predictors of response to CRT.^48^ The narrower the QRS duration, the higher is the degree of ventricular synchronization that can be obtained after pacing. In this study, we observed that HBP and LBBAP delivered a significantly narrower QRS duration compared with BVP. This may be explained by that HBP had the potential to capture the His‐bundles and contribute to the most effective ventricular resynchronization.^13^ Theoretically, LBBAP corrects the left bundle branch and leaves right bundle dyssynchronization, and may therefore have a longer QRS duration compared with HBP. However, our meta‐analysis found that there were no differences between the HBP and LBBAP groups. This may be explained by the fact that the conduction velocity in the Purkinje fibers is so rapid that there is almost no difference in the QRS width after HBP and LBBAP.^49^ Moreover, if the proximal LBBAP is performed, the paced QRS duration maybe not significantly longer compared with HBP. And the paced QRS duration can be further shortened during LBBAP by adjusting the AV delay or bipolar pacing to eliminate right bundle branch block pattern, which results in a nearly normal QRS complex. In contrast, BVP simply confers a mechanical synchronization rather than a physiologic synchronization; thus, it may not contribute to the full potential of CRT,^10^ which is why the QRS duration was longer than that of LBBAP. To the best of our knowledge, the long‐term results of the MADIT‐CRT study (7 years) highlighted the lack of benefit of CRT in nonspecific intraventricular conduction delay (NICD) patients compared with patients with LBBB.^50^ In the one included study of Upadhyay et al.,^33^ however, the crossover rates accounted for 50% from His‐CRT to BVP‐CRT due to NICD, which may affect the feasibility and outcomes between the HBP and BVP.

In theory, HBP and LBBAP confer physiologic pacing with a highly ventricular resynchronization; thus, there should be more LVEF improvement than BVP. We observed that all these resynchronization approaches had a significant improvement in LVEF in this meta‐analysis. Furthermore, the improvements of HBP and LBBAP are greater than those of BVP. Nevertheless, HBP and LBBAP do not have a large impact, as hypothesized. Previous studies showed that the absolute LVEF improvement of HBP ranges from 6% to 23%.^11^, ^14^, ^51^, ^52^ Similarly, the absolute LVEF improvement of LBBAP ranges from 16% to 24%.^21^, ^23^, ^34^, ^35^ However, different results were observed in different studies comparing the changes in LVEF between HBP/LBBAP and BVP. In some studies, no statistical significance was reached in LVEF improvement between LBBAP/HBP and BVP.^11^, ^21^, ^33^, ^34^, ^36^ In other studies, the LVEF improvement of HBP/LBBAP was significantly higher than that of BVP.^13^, ^23^, ^35^ The improvement in LVEF of HBP/LBBAP was inconsistent with the degree of narrowing of the QRS. These results can be explained by the small sample size, the nonrandomized study design, and the relatively short follow‐up period. Moreover, in addition to cardiac synchrony affecting the response to HBP/LBBAP, other variables may also be critically important to respond to CRT, such as age, sex, diabetes, PR interval, QRS morphology, myocardial ischemia, or scar.^53^ Further investigations should be done to provide *additional evidence* of the His‐Purkinje pacing for CRT response. With more methodological and clinical research and a better understanding of the features of the His‐Purkinje pacing, HBP/LBBAP would be more applicable as a supplement to BVP.

In addition to the clinical benefits and electrical synchrony, pacing parameters were *also important* in pacing treatments, such as pacing threshold and impedance. The early studies found that the pacing threshold of LBBAP was significantly lower than that of HBP,^35^, ^54^ which was even up to 2.75 V/1.0 ms in some cases.^33^ Our results were consistent with those of the previous studies that reported that HBP had a higher pacing threshold. The following reasons may explain these results. First, HB is covered with a fibrous sheath that is electrically nonconducting. Second, the HB is in a nondependent position, and orientation of the active fixation lead may influence the pacing thresholds.^55^ Furthermore, myocardial fibrosis and degeneration occur in the pacing area, and the pacing threshold increases after HBP *implantation*.^56^ Conversely, the LBBAP lead offers very low capture thresholds. This could be the result of a combination of factors. First, the LBB goes beneath the endocardium of the ventricular septum with a relatively large dimension and is surrounded by myocardium, thereby making it easier to capture.^49^ Second, the LBB lead targets the precise area just beyond the site of the conduction block.^17^, ^26^ Collectively, our results showed that LBBAP can achieve a comparable LV electrical and mechanical synchrony to HBP but with a lower pacing threshold; therefore, it might be superior to HBP for CRT.

### 4.1 Limitations

This meta‐analysis has several potential limitations. First, the small sample size may therefore possibly contribute to an underestimation of the accuracy of this study. Second, the lack of uniform criteria for LBBAP may influence its actual effects. The characteristics of the ECG and the EGM in the LBBAP procedure, such as stim‐LVAT, paced QRS morphology, and discrete component in the EGM, as the indirect criteria for LBB capture, were mainly used to distinguish LBBP from LVSP in previous study.^57^ Indeed, it was difficult to distinguish them accurately in some cases. Wu et al.^58^ currently proposed that retrograde His potential on the HBP lead and/or anterograde left conduction system potentials on the multielectrode catheter during LBBP were defined as the criteria for direct LBB capture, which could be used to distinguish LBBP from LVSP more accurately. Third, our included articles used different His or LV pulse width to detect the His or LV pacing threshold, which were 0.4 ms/0.5 ms/1.0 ms. Moreover, the average of the follow‐up duration of included articles ranged from 6 to 12.2 months, the potential long‐term outcomes and safety of these different types of CRT need to be investigated. Furthermore, the possible publication bias of changes in QRS duration should be taken into consideration *because* the study with *positive results* were *easier to* be reported. Last, the present study should use more indicators of clinical outcomes, such as left ventricular end‐diastolic diameter, blood B‐type natriuretic peptide, New York Heart Association classification, to evaluate the results. Unfortunately, very little data were reported for these outcomes, and network meta‐analyses were not possible. Despite the above limitations, this is the first NMA to provide the latest evidence of changes in LVEF, changes in QRS duration, and pacing threshold of BVP, HBP, and LBBAP delivered for CRT.

## 5 CONCLUSION

This is the first NMA that has analyzed the types of CRT and has demonstrated that LBBAP and HBP result in a greater LVEF improvement and a narrower QRS duration compared with BVP. Additionally, LBBAP was associated with a similar electromechanical resynchronization but lower pacing thresholds compared with HBP; therefore, it may offer advantages over HBP for CRT.

## CONFLICT OF INTERESTS

The authors declare no conflict of interest.

## AUTHOR CONTRIBUTIONS

Juan Hua and Chenxi Wang performed the meta‐analysis; Qiling Kong, Yichu Zhang, Qijun Wang, Ziyi Xiong were responsible for the statistical analysis; Jinzhu Hu, Juxiang Li, and Kui Hong provided editing assistance; and Qi Chen prepared the manuscript. All the authors have reviewed and agreed to this information before submission

## Supporting information

Supporting information.

## Data Availability

The data supporting this network meta‐analysis are from previously reported studies and datasets, which have been cited.
