# Supplementary material for: Comparative effects of left bundle branch area pacing, His bundle pacing, biventricular pacing in patients requiring cardiac resynchronization therapy: A network meta‐analysis
Source: Clin Cardiol. 2022 Feb 7;45(2):214–23. doi: 10.1002/clc.23784 (PMC8860481; doi:10.1002/clc.23784)
Supplement: Supplementary file 1 — Supporting information. [file CLC-45-214-s001.doc]

**Comparative effects of left bundle branch area pacing, His bundle pacing, biventricular pacing in patients requiring cardiac resynchronization therapy: A network meta-analysis**

**Authors:**

Juan Hua MD^1#^, Chenxi Wang MD^1#^, Qiling Kong MD^1^, Yichu Zhang MD^1^, Qijun Wang MD^1^, Ziyi Xiong MD^1^, Jinzhu Hu MD, PhD^1^, Juxiang Li MD, PhD^1^, Qi Chen MD, PhD^1*^, Kui Hong MD, PhD^1^

^#^Juan Hua and Chenxi Wang contributed equally to this paper.

**Affiliations:**

^1^ Department of Cardiology, The Second Affiliated Hospital of Nanchang University, Nanchang, Jiangxi 330006, P.R. China

***Correspondence:**

Professor Qi Chen, Department of Cardiology, The Second Affiliated Hospital of Nanchang University, 1 Minde Road, Nanchang, Jiangxi 330006, P.R. China. E-mail: [efycq@189.cn](mailto:efycq@189.cn).

This supplementary data provided the electronic search strategies, quality evaluation of RCTs, network plots for comparisons of outcomes, evaluation of inconsistency at global and local level, and publication bias of funnel plot.

**Supplementary**

**List of Supplementary Page**

**Supplementary Table S1:** Electronic search strategies 3~4

**Supplementary Figure S1：**Quality evaluation of included RCTs 5

**Figure S1-**A: Risks of bias graph

**Figure S1-**B: Risk of bias summary

**Supplementary Figure S2：**Network plots for comparisons 6

**Supplementary Figure S3：**Global inconsistency 7~8

**Figure S3-**A: Inconsistency for changes in LVEF improvement

**Figure S3-**B: Inconsistency for changes in QRS duration narrowing

**Figure S3-**C: Inconsistency for pacing threshold

**Supplementary** **Table S2-4**: Local inconsistency (Node-splitting) 9

**Table S2**: Inconsistency for changes in LVEF improvement

**Table S3**: Inconsistency for changes in QRS duration narrowing

**Table S4**: Inconsistency for pacing threshold

**Supplementary** **Figure S4**: Publication bias of funnel plot 10~11

**Figure S4-**A: funnel plot for changes in LVEF improvement

**Figure S4-**B: funnel plot for changes in QRS duration narrowing

**Figure S4-**C: funnel plot for pacing threshold

| **Search** | **Query** |
| --- | --- |
| **PubMed** |  |
| #1 | Cardiac resynchronization therapy OR CRT |
| #2 | Biventricular pacing OR BiV pacing OR BVP OR BIVP |
| #3 | His bundle pacing OR HBP |
| #4 | Left bundle branch pacing OR LBBP OR Left bundle branch area pacing OR LBBAP OR Left ventricular septal pacing OR LVSP |
| #5 | #1 and #2 |
| #6 | #1 and #3 |
| #7 | #1 and #4 |
| #8 | #5 and #6 |
| #9 | #5 and #7 |
| #10 | #6 and #7 |
| #11 | #8 or #9 or #10 |
| **Embase** |  |
| #1 | (Cardiac resynchronization therapy) OR (CRT) |
| #2 | (Biventricular pacing) OR (BiV pacing) OR (BVP) OR (BIVP) |
| #3 | (His bundle pacing) OR (HBP) |
| #4 | (Left bundle branch pacing) OR (LBBP) OR (Left bundle branch area pacing) OR (LBBAP) OR (Left ventricular septal pacing) OR (LVSP) |
| #5 | #1 and #2 |
| #6 | #1 and #3 |
| #7 | #1 and #4 |
| #8 | #5 and #6 |
| #9 | #5 and #7 |
| #10 | #6 and #7 |
| #11 | #8 or #9 or #10 |
| **Web of Science** |  |
| #1 | (Cardiac resynchronization therapy) OR (CRT) |
| #2 | (Biventricular pacing) OR (BiV pacing) OR (BVP) OR (BIVP) |
| #3 | (His bundle pacing) OR (HBP) |
| #4 | (Left bundle branch pacing) OR (LBBP) OR (Left bundle branch area pacing) OR (LBBAP) OR (Left ventricular septal pacing) OR (LVSP) |
| #5 | #1 and #2 |
| #6 | #1 and #3 |
| #7 | #1 and #4 |
| #8 | #5 and #6 |
| #9 | #5 and #7 |
| #10 | #6 and #7 |
| #11 | #8 or #9 or #10 |
| **The Cochrane Library** |  |
| #1 | (Cardiac resynchronization therapy) OR (CRT) in All Text |
| #2 | (Biventricular pacing) OR (BiV pacing) OR (BVP) OR (BIVP) in All Text |
| #3 | (His bundle pacing) OR (HBP) in All Text |
| #4 | (Left bundle branch pacing) OR (LBBP) OR (Left bundle branch area pacing) OR (LBBAP) OR (Left ventricular septal pacing) OR (LVSP) in All Text |
| #5 | #1 and #2 |
| #6 | #1 and #3 |
| #7 | #1 and #4 |
| #8 | #5 and #6 |
| #9 | #5 and #7 |
| #10 | #6 and #7 |
| #11 | #8 or #9 or #10 |

Table S1: Electronic search strategies determined on 11^st^ July.2021

**Figure S1** Quality evaluation of included RCTs
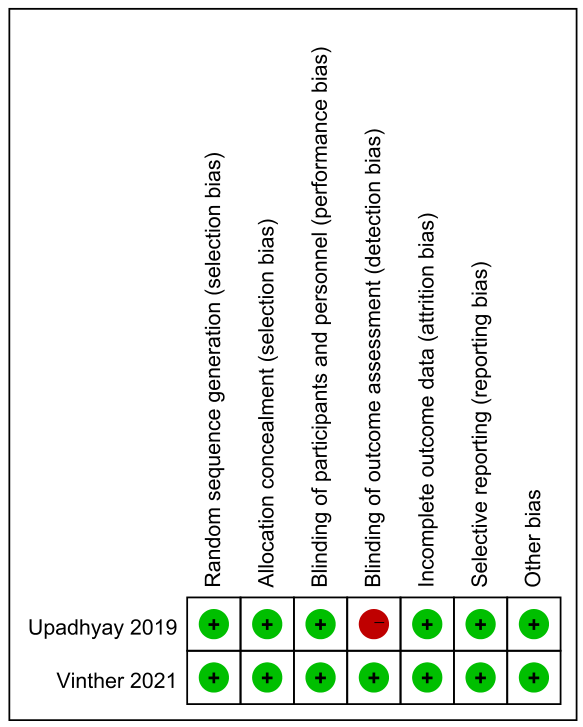


**Figure S1-**A**:** Risk of bias summary**.** The qualities of the included two RCTs, which were evaluated using the Cochrane bias risk assessment tool provided by the Review Manager. Different colors (green, yellow, and red) were used in the figure to indicate low-risk, unclear, and high-risk bias, respectively.


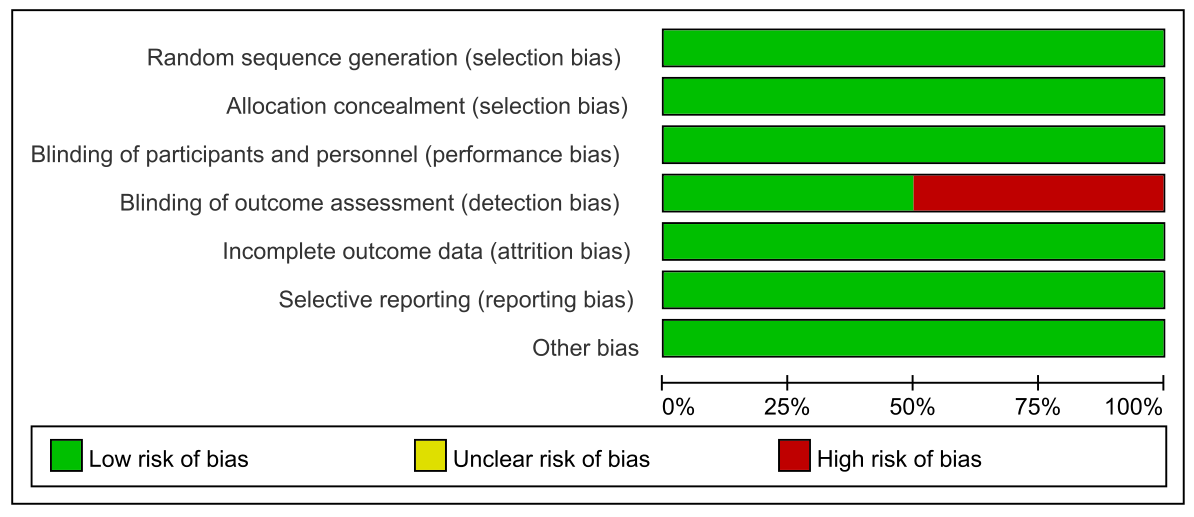


**Figure S1-**B：**Risks of bias graph**. The percentage of each level and level of each specific study item is presented. Abbreviations: RCT, randomized controlled trials.

**Figure S2** Network plots for comparisons of outcomes


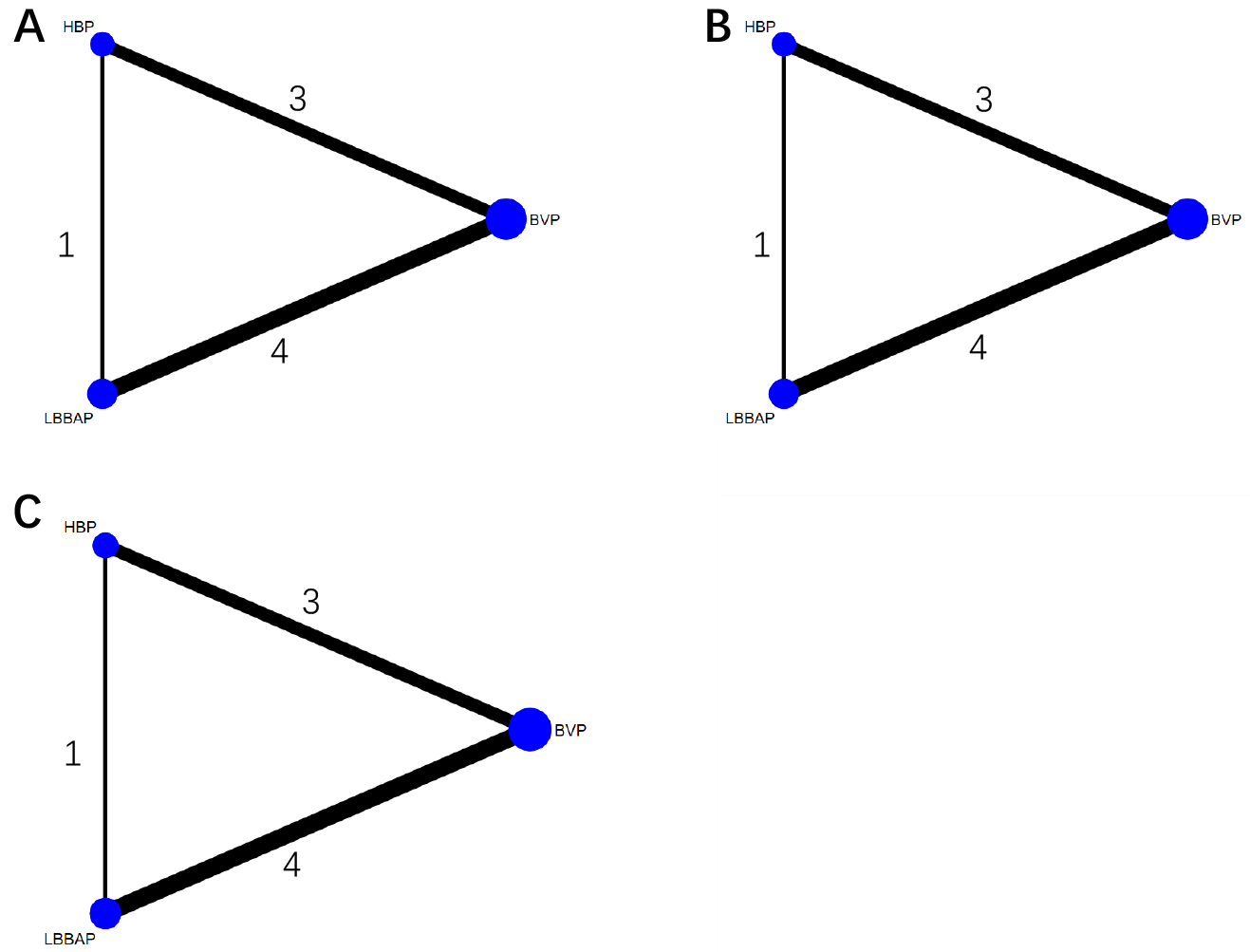


**Figure S2.** Network plots for comparisons of changes in LVEF improvement (A), changes in QRSd narrowing (B), and pacing threshold (C) between BVP, HBP and LBBAP. The sizes of the nodes represented the number of patients on treatment. The widths of the lines represented the number of patients in trials with direct comparison between the nodes. The numbers on the lines represented the number of trials with direct comparisons. LVEF, left ventricular ejection fraction; QRSd, QRS duration; BVP, biventricular pacing; HBP, His bundle pacing; LBBAP, left bundle branch area pacing.

**Figure S3** Global inconsistency


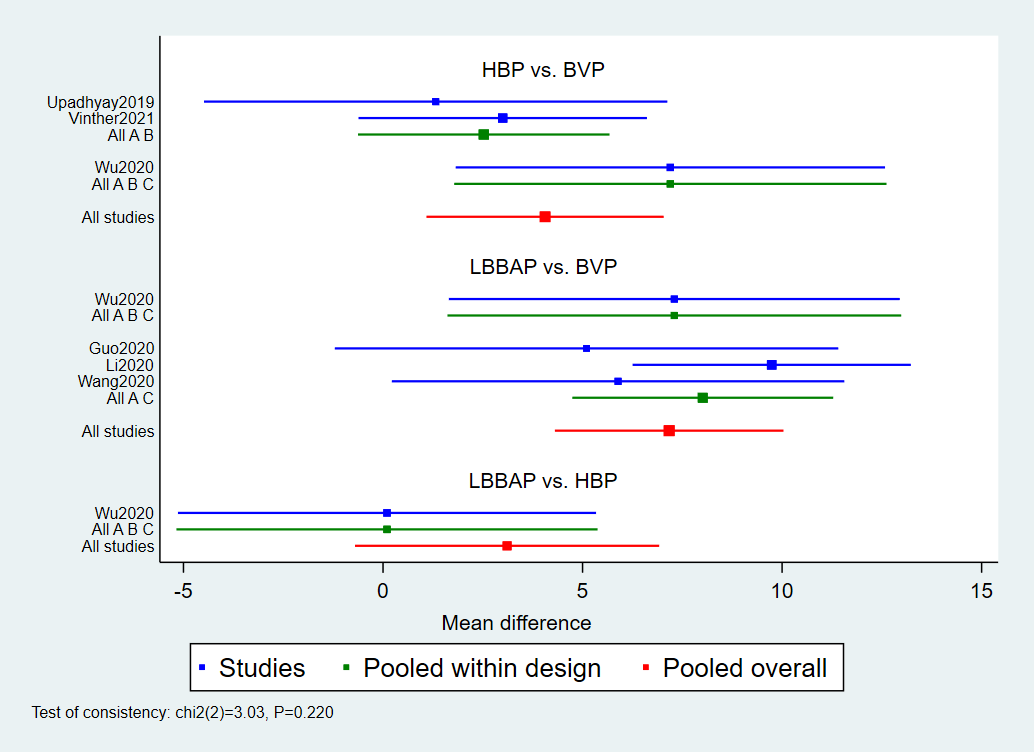


**Figure S3-**A: Inconsistency for changes in LVEF improvement.


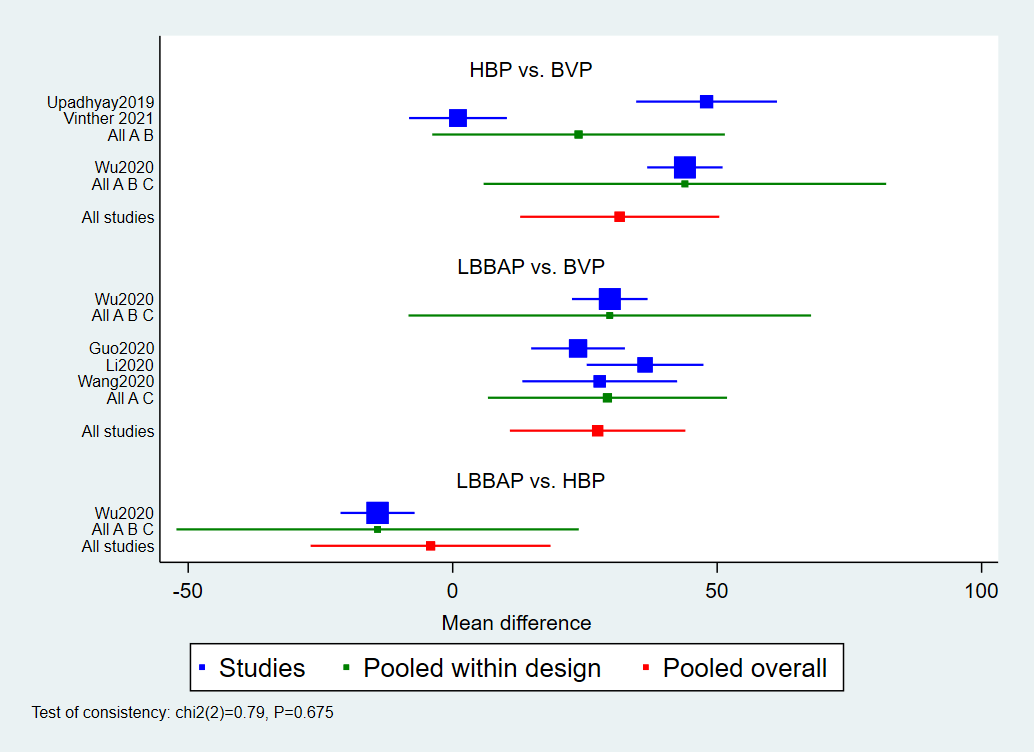


**Figure S3-**B: Inconsistency for changes in QRS duration narrowing.


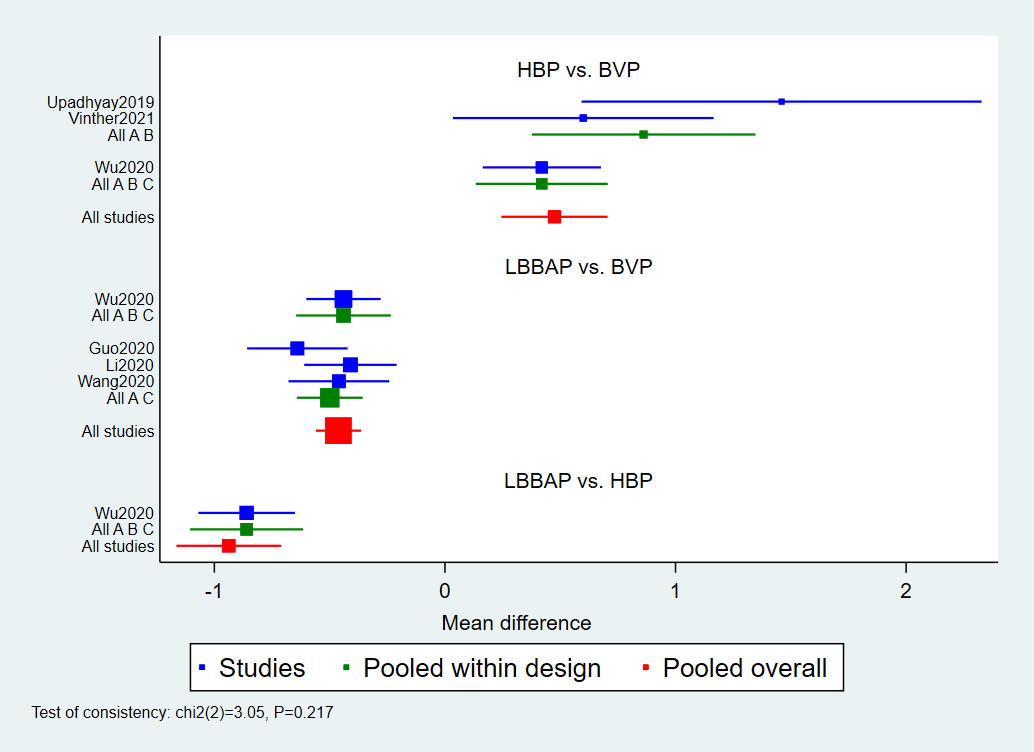


**Figure S3-**C: Inconsistency for pacing threshold.

**Table S2-4**. Local Inconsistency (Node-splitting)

| Side | Direct | | | Indirect | | | | Difference | | | | P>|z| |
| --- | --- | --- | --- | --- | --- | --- | --- | --- | --- | --- | --- | --- |
|  | Coef. | Std. Err. |  | | Coef. | Std. Err. |  | | Coef. | Std. Err. |  |  |
| A B * | 3.726691 | 1.544126 |  | | 8.676337 | 6.320412 |  | | -4.949646 | 6.592974 |  | 0.453 |
| A C * | 7.898344 | 1.238095 |  | | -2.06649 | 5.552584 |  | | 9.964834 | 5.701569 |  | 0.081 |
| B C | -.0058818 | 2.66888 |  | | 5.425075 | 2.078145 |  | | -5.430956 | 3.387082 |  | 0.109 |

**Table S2**: Inconsistency for changes in LVEF improvement. A, BVP; B, HBP; C, LBBAP

| Side | Direct | | | Indirect | | | | Difference | | | | P>|z| |
| --- | --- | --- | --- | --- | --- | --- | --- | --- | --- | --- | --- | --- |
|  | Coef. | Std. Err. |  | | Coef. | Std. Err. |  | | Coef. | Std. Err. |  |  |
| A B * | 30.72287 | 11.041 |  | | 43.04297 | 39.43186 |  | | -12.3201 | 40.95284 |  | 0.764 |
| A C * | 29.37097 | 8.512027 |  | | -11.00161 | 37.54786 |  | | 40.37258 | 38.50311 |  | 0.294 |
| B C | -14.20131 | 17.50179 |  | | 4.675238 | 16.46481 |  | | -18.87655 | 24.02936 |  | 0.432 |

**Table S3**: Inconsistency for changes in QRS duration narrowing. A, BVP; B, HBP; C, LBBAP

| Side | Direct | | | Indirect | | | | Difference | | | | P>|z| |
| --- | --- | --- | --- | --- | --- | --- | --- | --- | --- | --- | --- | --- |
|  | Coef. | Std. Err. |  | | Coef. | Std. Err. |  | | Coef. | Std. Err. |  |  |
| A B * | .5426241 | .1483686 |  | | .3240693 | .2520112 |  | | .2185548 | .2847191 |  | 0.443 |
| A C * | -.4767813 | .0496954 |  | | .4638158 | .5298989 |  | | -.9405971 | .5361248 |  | 0.079 |
| B C | -.893928 | .1240443 |  | | -1.179084 | .2248916 |  | | .2851558 | .2442448 |  | 0.243 |

**Table S4:** Inconsistency for pacing threshold. A, BVP; B, HBP; C, LBBAP

**Figure S4**. Publication bias of funnel plot


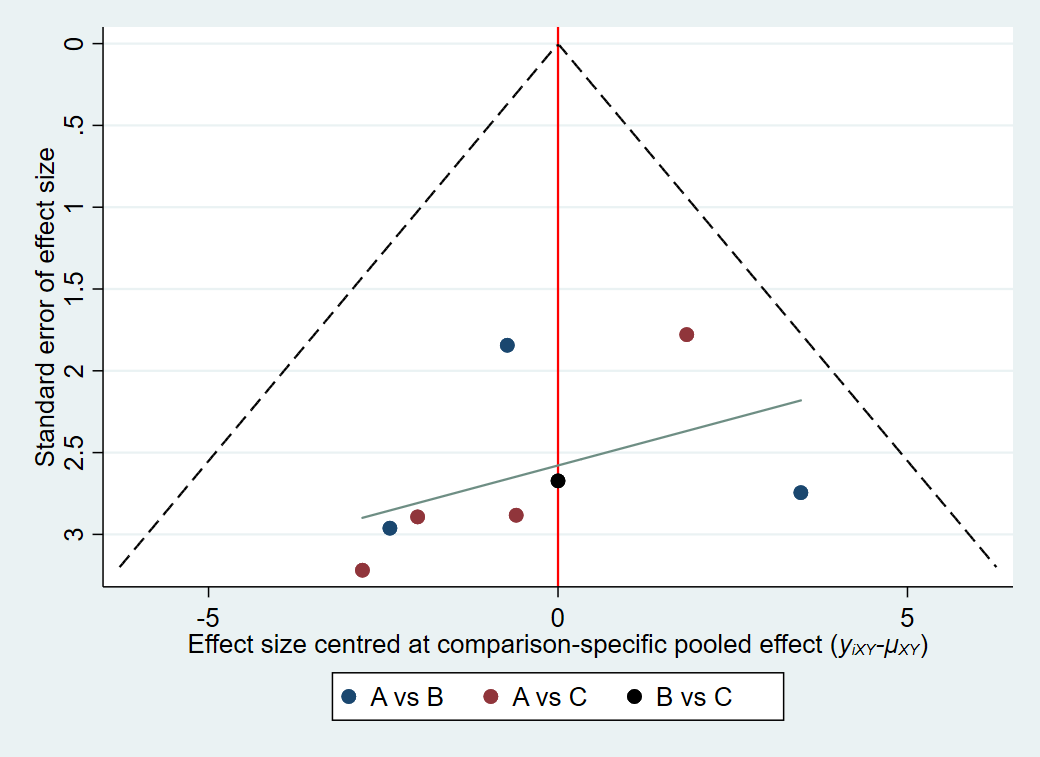


**Figure S4**-A: funnel plot for changes in LVEF improvement. (A, BVP; B, HBP; C, LBBAP)


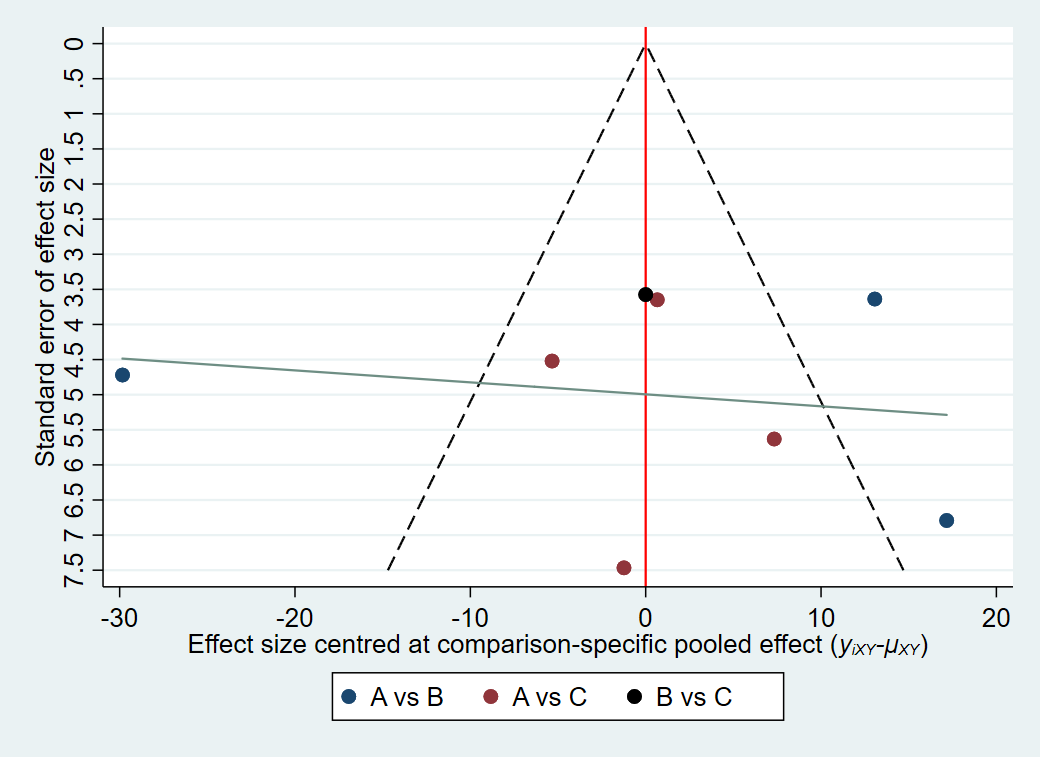


**Figure S4**-B: funnel plot for changes in QRS duration narrowing. (A, BVP; B, HBP; C, LBBAP)


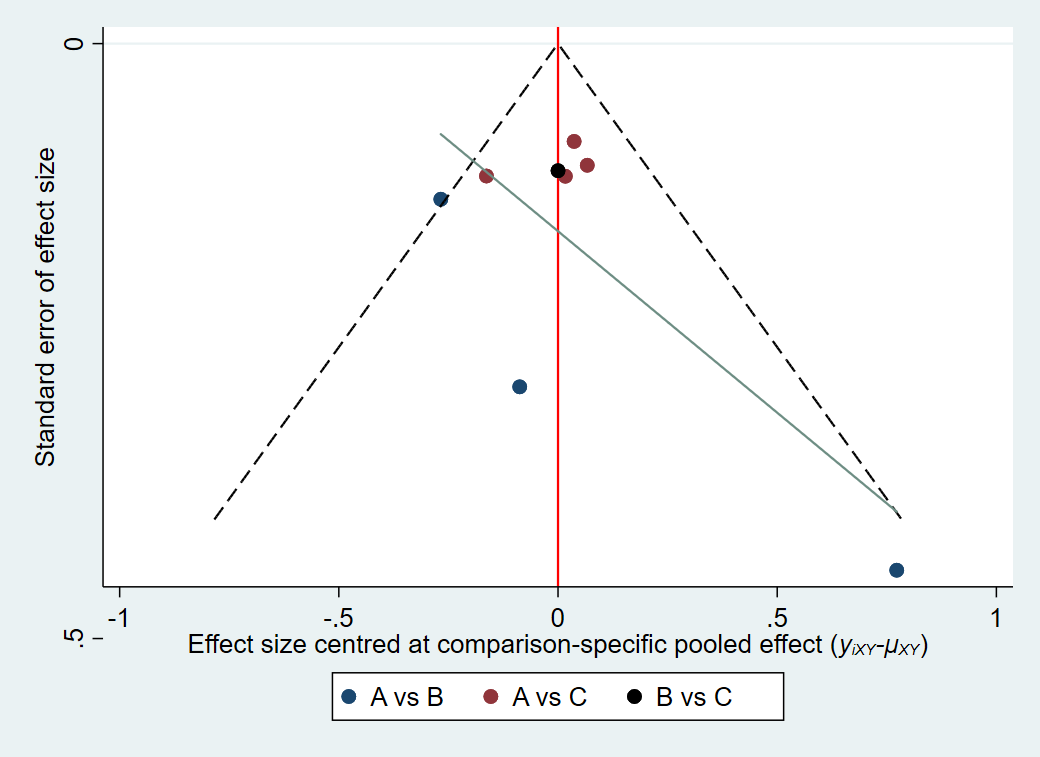


**Figure S4**-C: funnel plot for pacing threshold. (A, BVP; B, HBP; C, LBBAP)
